# Submillimetre Network Formation by Light-induced Hybridization of Zeptomole-level DNA

**DOI:** 10.1038/srep37768

**Published:** 2016-12-05

**Authors:** Takuya Iida, Yushi Nishimura, Mamoru Tamura, Keisuke Nishida, Syoji Ito, Shiho Tokonami

**Affiliations:** 1 Department of Physical Science, Graduate School of Science, Osaka Prefecture University, Sakai, Osaka 599-8570, Japan; 2 Nanoscience and Nanotechnology Research Center, Osaka Prefecture University, 1-2, Gakuencho, Nakaku, Sakai, Osaka 599-8570, Japan; 3 Department of Applied Chemistry, Graduate School of Engineering, Osaka Prefecture University, 1-2, Gakuencho, Nakaku, Sakai, Osaka 599-8570, Japan; 4 Division of Frontier Materials Science, Graduate School of Engineering Science, Osaka University, 1-3, Machikaneyama, Toyonaka, Osaka 560-8531, Japan

## Abstract

Macroscopic unique self-assembled structures are produced via double-stranded DNA formation (hybridization) as a specific binding essential in biological systems. However, a large amount of complementary DNA molecules are usually required to form an optically observable structure via natural hybridization, and the detection of small amounts of DNA less than femtomole requires complex and time-consuming procedures. Here, we demonstrate the laser-induced acceleration of hybridization between zeptomole-level DNA and DNA-modified nanoparticles (NPs), resulting in the assembly of a submillimetre network-like structure at the desired position with a dramatic spectral modulation within several minutes. The gradual enhancement of light-induced force and convection facilitated the two-dimensional network growth near the air-liquid interface with optical and fluidic symmetry breakdown. The simultaneous microscope observation and local spectroscopy revealed that the assembling process and spectral change are sensitive to the DNA sequence. Our findings establish innovative guiding principles for facile bottom-up production via various biomolecular recognition events.

The helical structure of DNA was identified in 1953^1^. Subsequently, gene analysis methods, such as the Sanger method and PCR, were developed^2^,^3^ and the human genome sequencing was completed in the early 21^st^ century^4^. Recently, highly sensitive and rapid methods for DNA detection are required in the healthcare and food industries^5^,^6^,^7^,^8^,^9^. Particularly, potential applications of functional self-assembled structures via DNA have received significant attention in the field of information technology, photonics, and biomedicine^10^,^11^,^12^. The enhanced near-field in a nanogap between gold nanoparticles (AuNPs) fixed on a micropillar fabricated using DNA origami techniques^13^ was used for the detection of small amounts of DNA by fluorescent imaging^14^. These methods can observe a small amount of DNA, but are complex, time-consuming, and expensive since fluorescent dyes and advanced optical systems are required. DNA base complementarity has been exploited to create self-assembling macroscopic super-lattice structures of terminally thiolated single-strand DNA molecules bound to AuNPs and complementary DNA^15^,^16^,^17^,^18^,^19^. These structures are utilized for label-free detection of 5 fmol of sample DNA, via the measurement of electric current change in assembled probe nanoparticles (DNA-modified AuNPs)^20^, where each assembled structure was smaller than 100 nm and observed by FE-SEM. The ability to remotely and physically control the specific binding of probe NPs and target DNA should enable a dramatic expansion in the range of applications of hybridization. For example, the exploitation of the “light-induced force (LIF)” that arises from the mechanical interaction between light and matter^21^,^22^,^23^,^24^ should enable control of the dynamics and trapping of small objects in a non-contact-based and non-destructive manner using laser irradiation, whereas the control of NP dynamics remains challenging. Another report described the rapid assembly of small objects by “light-induced convection (LIC)” of high-density metallic NPs initially fixed on micro beads^25^, wherein a macroscopic bubble was simultaneously generated by the strong infrared photothermal effect. However, a more moderate assembly process with less heat is desired for the optical control of DNA hybridization since a binding process with biomolecular recognition is often fragile. The spectral broadening by plasmonic superradiance and redshift as collective phenomena of LSPs^26^ through the soft assembling process of dispersed metallic NPs by LIF would facilitate the gradual enhancement of photothermal effect and LIC.

Here, based on such a strategy, we aim at the development of the guiding principle for “*Light-induced Acceleration of DNA hybridization*” mediated by NPs to form a macroscopic network stably. For this purpose, we try to enhance the photothermal effect by exploiting the collective phenomena of LSPs via the assembly process of low-density probe NPs and target substances for the moderate enhancement of LIC in addition to LIF with molecular recognition. This strategy provides the opportunity for optical control of molecular recognition mechanisms and the development of unconventional nanofabrication methods. Particularly, we have developed our original theoretical method “Light-induced Molecular Recognition Metropolis Method (LMRM)” to clarify the role of LIF and molecular recognition in the assembly process of probe NPs and target molecules. And, we experimentally investigated the laser-induced dynamics and hybridization of DNA-modified probe NPs and target DNA by the simultaneous microscopic observation and local spectroscopy near the air-liquid interface. There, the symmetry in the optical field and the liquid flow was broken, the positive feedback under the nonequilibrium process by the synergetic combination of LIF and LIC would play crucial roles in an assembling process of macroscopic network.

## Results

### Light-induced Acceleration

**Figure 1:**
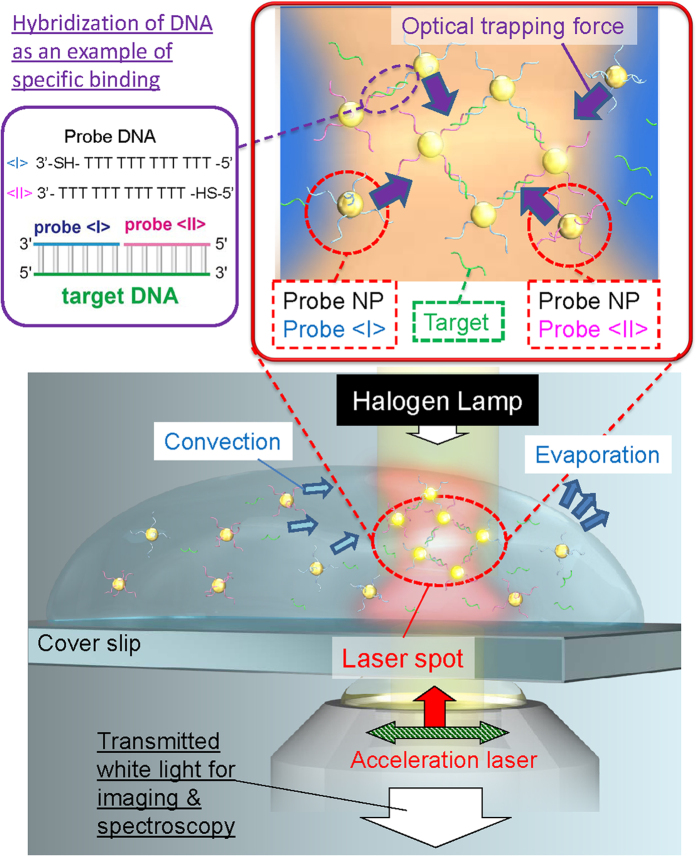
Schematic illustration of accelerated double-stranded DNA formation with optically-assembled gold nanoparticles (AuNPs) as probes. An infrared laser was focused near the air-liquid interface, where probe NPs and target DNA were transported by convection flow, trapped by light-induced force (LIF), and densely concentrated during the evaporation process.

**Figure 2:**
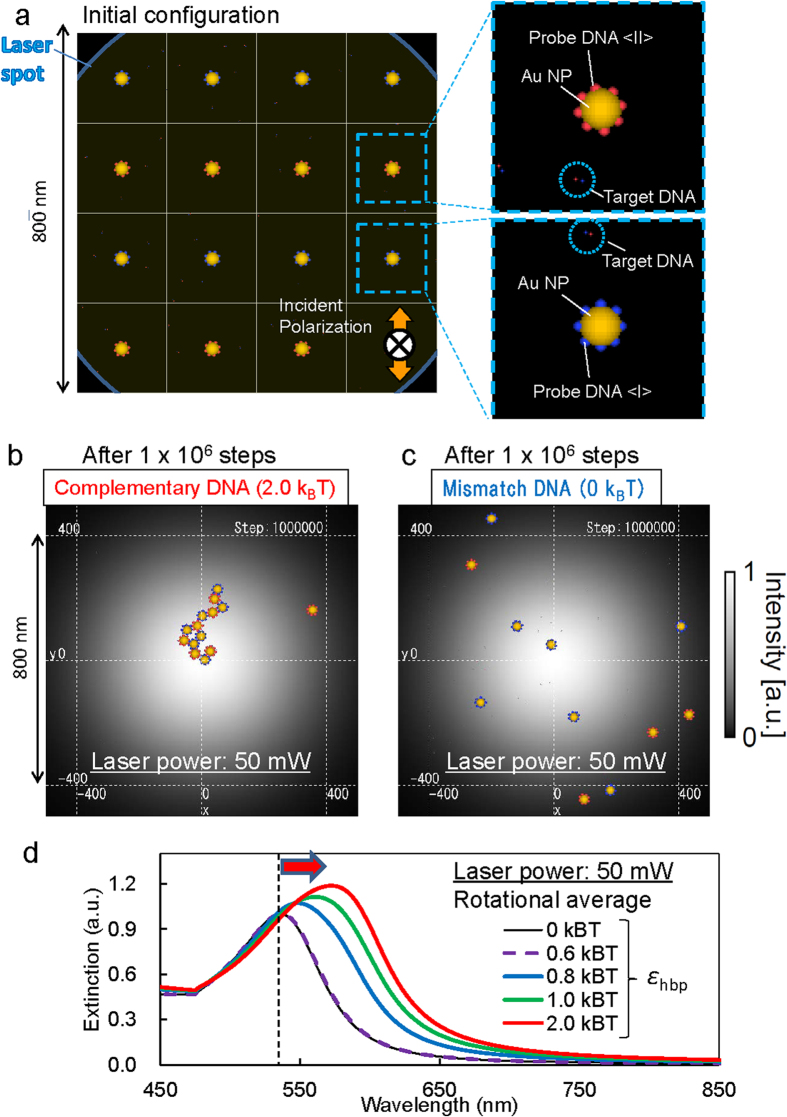
Simulation of the optical acceleration of DNA hybridization with probe AuNPs by the Light-induced Molecular Recognition Metropolis Method (LMRM). (**a**) Model for calculation: the total number of Monte Carlo steps is 10^6^. Final configurations for (**b**) complementary and (**c**) mismatched DNA (see also Supplementary movies S1 and S2). In (**b,c**) the wavelength was 1,064 nm, diameter of laser spot 1 μm, AuNP diameter 30 nm, and input power 50 mW at the focused area. (**d**) Calculated extinction spectra of assembled NPs with complementary DNA or mismatched DNA for different complementarity related with binding energies. The vertical dashed line indicates the initial LSP peak position, and the red arrow indicates the peak shift under light-induced hybridization depending on *ε*_hbp_.

Figure 1 describes the main concept of light-induced acceleration of DNA hybridization mediated by interfacial symmetry breakdown for optical field and liquid flow in this study. Probe NPs <I> and <II> are modified with 3′-terminally thiolated DNA and 5′-terminally thiolated DNA, respectively, in order to form the assembled structure via the hybridization with target DNA. In order to characterize the early steps of optical acceleration of DNA double-strand formation by LIF, we analysed the hybridization of probe NPs and target DNA using our developed theoretical method LMRM [see Methods]. This theoretical method has enabled the evaluation of energetically metastable states under LIF and molecular recognition based on the stochastic method^26^,^27^, in view of the self-consistent interaction between optically-induced polarizations of LSP in respective probe NPs and DNA hybridization (Fig. 2). As shown in Fig. 2b, an assembled structure was optically trapped near the focal point in the case of complementary DNA (Supplementary movies S1). However, as the optical trapping force (gradient force) exerted on an isolated AuNP is weak (Supplementary movies S2), probe NPs and mismatched target DNA molecules freely diffused by Brownian motion (as shown in Fig. 2c). Furthermore, we demonstrated that, following assembly by LIF, the calculated extinction spectrum exhibits a prominent red shift and spectral broadening owing to plasmonic superradiance, as DNA complementarity increases with the interaction energy per base pair comprising the double stranded DNA (*ε*_hbp_) (Fig. 2d). This indicates that the optical absorption gradually increases to generate higher heat during an assembling process under the irradiation of an infrared laser.

### Rapid macroscopic network formation

An infrared laser of 43 mW after transmission from the objective lens (wavelength, 1064 nm) was focused near the air-liquid interface and the edge of the droplet for accelerating the hybridization of the probe and target DNA by LIF and LIC [See Methods]. This power was carefully selected as the optimal setting. Higher laser power suppresses the growth of assembled structures due to the generation of too much amount of heat while probe NPs cannot be trapped at lower laser power. As a result of evaporation-induced condensation, the edge of the droplet contains more NPs and target DNA; this was confirmed by optical microscopy during the natural drying process. In addition, the probability of collision of probe NPs and target DNA molecules was increased. DNA molecules (24 bases in length) of 5 different sequences (AAAA, AATT, TATA, TTTT, and RAN-C) were used as targets (detailed information is provided in Methods).

**Figure 3:**
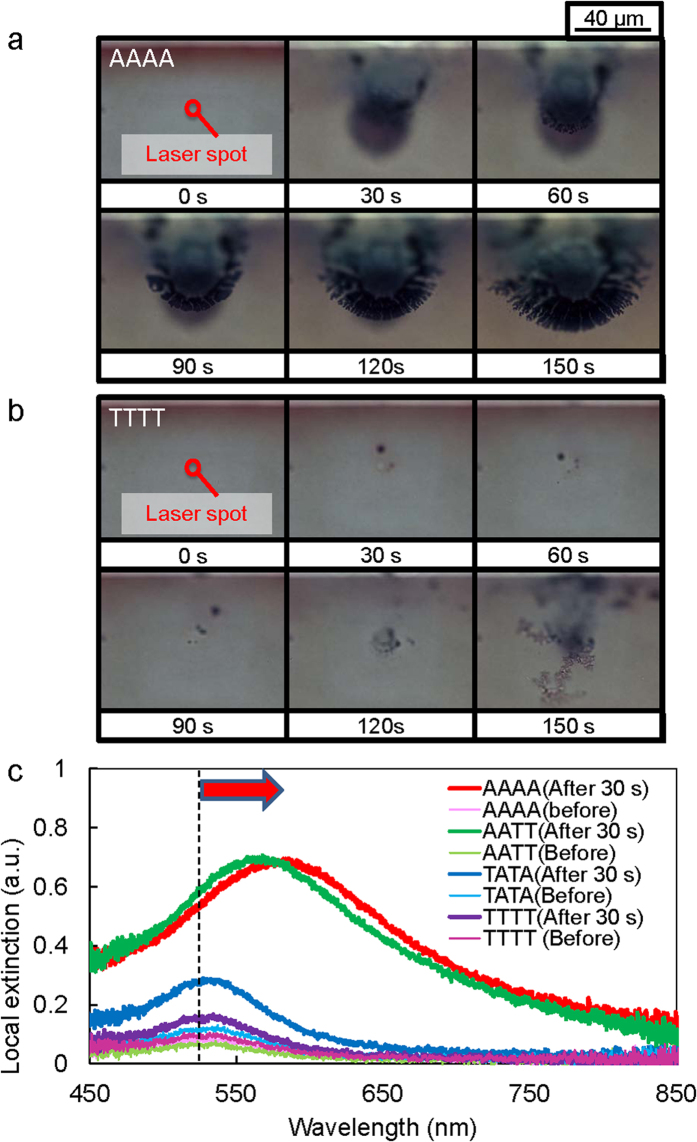
Experimental demonstration of light-induced acceleration of DNA hybridization. (**a**) The assembly of target DNA and probe NPs was monitored at 30-s intervals using a CCD camera (see also Supplementary movie S3). Laser irradiation was initiated after diffusion of the probe NPs and target DNA. The dispersed liquid of complementary DNA (AAAA; 100 pM) in phosphate buffer as the target was added to a mixed suspension of probe NP <I> and <II>. Recording was initiated simultaneously with laser irradiation. (**b**) The same procedure (as in **a.)** was performed for mismatched DNA (TTTT; 100 pM) (Supplementary movie S6). Differences in behaviour were observed according to the base sequence of the target DNA (AATT, TATA) (Supplementary movies S4–S5). (**c**) Local extinction spectra of the assembled structure of probe NPs and various target DNA after 30-s laser irradiation, for complementary DNA (AAAA) and mismatched DNA (AATT, TATA, TTTT; see also optical images for each sequence in Fig. S1). The spectra prior to laser irradiation are also shown.

The mixture of probe NPs <I> and <II> with T-sequence DNA (PrT) and dilute complementary DNA (AAAA, 100 pM) in the substrate was irradiated with the first laser for 2.5 min, and diffusion was allowed to proceed for 5 min (further detail is provided in Methods). Immediately, after the irradiation with the second laser, a macroscopic network-like assembled structure was formed near the air-liquid interface (Fig. 3a). However, macroscopic assembly with 100 pM of AAAA did not occur by natural hybridization, even after 3 h (Fig. S3), while 1 μM of AAAA shows macroscopic assembly (Fig. S2). Also, when the target DNA was a perfect mismatch, the macroscopic network did not grow during the observation period (Fig. 3b). On the other hand, as shown in the theoretical result of Fig. 2b, the assembled structure was oriented parallel to the polarization of light when produced by LIF, where the attractive inter-object LIF parallel to the light polarization arises between induced polarizations of LSPs in probe NPs. Such an attractive force facilitates the formation of an elongated structure along the light polarization as shown in Fig. 2b. This means that the experimentally observed radial network structure cannot be explained with LIF alone. The differences between the theoretical and experimental data indicate that LIC plays an important role in the formation of the macroscopic assembled structure. We additionally confirmed that the assembly process was depending on the base sequence of the target DNA (AAAA, AATT, TATA, or TTTT) even for the same probe NPs of PrT, as shown in Fig. S1 and Supplementary movies S3–S6. The observed base-sequence dependence was investigated by examining the local extinction spectra for each target DNA sequence, both before and after 30-s laser irradiation, and it was found that the spectral change appeared to differ greatly by sequence (Fig. 3c). This result implied that higher complementarity induces larger spectral shifts which is consistent with theoretical results, as shown in Fig. 2d.

### DNA-sequence dependence

**Figure 4:**
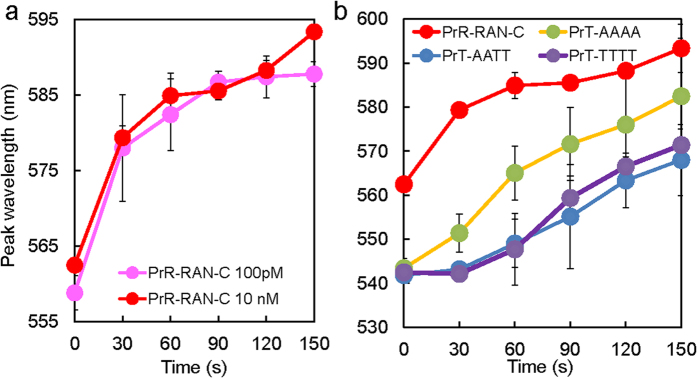
Dependence of optical acceleration on the DNA sequence. (**a**) Peak wavelength of localized surface plasmon (LSP) of the assembly over time for random sequences of DNA at various concentrations (100 pM and 10 nM) (**b**) Peak wavelength of LSP of the assembly over time for various DNA sequences at the same concentration (10 nM); for each curve, the average of three time measurements was taken. Vertical bars indicate the standard deviation of the peak wavelength (see also the optical image and spectrum for natural hybridization, without laser, for each sequence; Figs S4 and S5).

In order to visualize the sequence-dependent local spectral change during the formation of the network-like structure, the time dependence of the LSP resonance wavelength was plotted under single-laser irradiation with different spot heights (Fig. 4) [See Methods for more detail]. The spot height was set to several μm higher than the value used in Fig. 3 in order to slightly extend the spot area on the air-liquid interface for illuminating more probe NPs and target DNA. By changing concentrations of complementary target DNA and random probe (PrR), the peak shift becomes prominent after 150 s at a higher concentration (10 nM). Therefore, in the case of optical acceleration for PrT, a target DNA of 10 nM concentration (AAAA, AATT, TTTT) was used for the time-dependence experiment in order to increase reproducibility (Fig. 4b). The assembly was more prominent and spectral shift for PrR with the target RAN-C was larger than that of PrT with the target AAAA.

## Discussion

We attempt to illustrate the observed phenomena in Fig. 3. The mixture was warmed by laser absorption of the small aggregates of probe NPs trapped by LIF at the tail of red-shifted LSP resonance (Fig. 2d, Fig. 3c), and gentle convection flow was gradually enhanced. Also, the spectral modulation provided strong radiation pressure (dissipative force as a component of LIF) which pushed the NPs into the optical path towards the air-liquid interface. The temperature was estimated to be lower than the melting temperature of double-stranded DNA even during laser irradiation (*T*_m_ = 53 °C and 70 °C for 24 bases in the present T-sequence DNA and random-sequence DNA, respectively). LIC facilitates the transport of the probe NPs and target DNA toward the focal point of the laser, and leads to their condensation. During the gentle LIC step, in addition to a gradient force, attractive inter-object LIF was additionally exerted between the probe NPs. The probes were attracted to the region of highest light intensity, leading to hybridization and nucleation. Therefore, the probability of intermolecular collisions is enhanced, and the macroscopic assembling via hybridization can be optically accelerated. Since the gradient force is proportional to the volume, a large aggregate with a large volume can be strongly trapped. The estimated gradient force exerted on a single 30-nm AuNP ranged from several fN to several tens of fN under the assumed laser power^22^,^26^. Therefore, the generation of a sufficiently strong gradient force requires the assembly of more than 10 NPs, the total volume of which is 10 times larger than that of a single NP, for stable optical trapping using LIF. LIC is additionally considered to accelerate the assembly process. As the assembled structure grows while being trapped by the LIF, the LIC may be enhanced dramatically as a result of the increase in the volume of the heat source. This process provides the positive feedback required to drive the growth of the assembled structure under light-induced non-equilibrium conditions.

Based on the initial concentration, the estimated amount of target DNA was approximately 5 zmol in the photometric area for local extinction, which is enclosed within a 15-μm circle (Fig. S7). Therefore, we estimated that zmol-level complementary DNA contributes to macroscopic assembly formation. We then estimated the number of target DNA molecules contributing to the assembled macroscopic radial structure extending from the laser spot at 150 s (Fig. 3a). The radial structure consists of approximately 40 chain-like structures, with each structure about 25 μm long. Each pair of probe NPs was assumed to be connected by a single target DNA molecule, and 800 probe NPs by a similar number of target DNA molecules. Accordingly, 32,000 target DNA molecules were estimated to be contained within the assembled structure that extends in two dimensions along the air-liquid interface, representing 1/4 of the total number of target DNA molecules in the observed region. This estimation indicates that complementary DNA at the zmol level (~10^2^–10^3^ DNA molecules) became a trigger of the macroscopic assembling phenomena within a few minutes.

Furthermore, the results in Fig. 4 show the possibility to control the optical spectrum of macroscopic assembly of probe NPs and target DNA, in a DNA sequence-dependent manner, by laser-induced acceleration of molecular recognition even with a small amount of DNA. This means that a broadband optical antenna exhibiting tunable plasmonic superradiance^26^ can be created by light-induced DNA hybridization. The RAN-C sequence showed the most dramatic spectral change, followed by PrT and AAAA. The difference was attributed to the difference between the number of hydrogen bonds present in G ≡ C pairs and T = A pairs. This result indicates that increasing complementarity of DNA sequence was clearly associated with larger assembled structures. As shown in Figs S4 and S5, large network-like assembled structures and spectral changes can be observed for PrR with RAN-C and PrT with AAAA during several tens minutes in the absence of laser irradiation even when very high concentrations (1 μM) of target DNA with different sequences were used as comparison experiments.

In conclusion, the present work introduces the “Light-induced Acceleration of Molecular Recognition” strategy, which may be utilised in numerous techniques involving the production of network-like optical antenna, the control of various molecular recognition processes, not only DNA hybridization, as in this work, but also antigen-antibody reactions, sugar-lectin binding, and cell control via intracellular lasing^28^,^29^,^30^. These techniques will provide innovative methods in wide fields not only bottom-up nanofabrication, but also molecular cell biology, medical science, food inspection, and environmental monitoring.

## Methods

### Light-induced acceleration and local spectrum measurement

The continuous infrared laser light (1064 nm) for optical trapping was added to the inverted optical microscope (Eclipse Ti−U; Nikon, Japan) using a back port adapter (LMSAD-NI-BP; Sigma-Koki, Japan). The trapping laser was focused to a ∼1 μm diameter spot under a 100 × oil immersion objective lens (numerical aperture = 1.30). The input laser power (0.20 W at the light source) was reduced to approximately 21.7% (43 mW) after the objective, as measured by a power detector (UP17P-6S−H5 with TUNER; Gentec Electro-Optics, Canada). Sample-dispersed liquids (5.0 μL of 1.0 nM probe NP <I>, 5.0 μL target DNA in phosphate buffer, and 5.0 μL of 1.0 nM probe NP <II> in a total volume of 15.0 μL) were serially dropped onto the coverslip (thickness ∼0.17 mm) on the sample stage. The laser spot was set to 37.5 μm from the edge of the droplet on the substrate, and to 24 μm above the surface of the cover slip for Fig. 3, and 30 μm above the surface of the cover slip for Fig. 4 without correction for refractive index near the air-liquid interface. The optical trapping and assembly process was recorded (transmission image) during the laser irradiation under bright field conditions using a charge-coupled device (CCD) camera with a frame rate of 15 frames/s.

In Fig. 3, 5 minutes after complete droplet extension and diffusion, the first laser was applied for 2.5 min and turned off for 0.5 min to allow assembly of the probe NPs and target DNA into a small core structure. After further 5 minutes, the second laser (acceleration laser) was applied at the horizontally opposite side of the droplet in the same manner; this process was recorded with the CCD camera. In Fig. 4, only a single laser irradiation was performed for 3 minutes at 13 minutes after complete droplet extension and diffusion, which corresponds to the timing of the 2nd laser irradiation in Fig. 3. Local extinction spectra around the laser spot were observed every 30 s with a miniature fiber-optic spectrometer [USB4000 (Grating#3) SLIT-25, Ocean Optics, USA] in the both cases. As shown in Fig. 4, the spectral change in the case of mismatched target DNA (AATT and TTTT) was much smaller than the complementary case, which is consistent with the preliminary experiments without laser irradiation in Figs S2–S5 (we have also confirmed that AuNPs were not assembled in the similar experiment without DNA).

### Modification of Probe DNA on Au Nanoparticles

Modification of oligonucleotide to AuNPs^15^,^16^,^20^: 3.61 μM thiolated oligonucleotide was incubated in 1000 μL of a 30-nm AuNP (synthesized by a citrate reduction^31^) solution at 298 K for 16 h, where poly-T probe (PrT): 5′-TTT TTT TTT TTT-3′-(CH_2_)_6_-SH for probe <I>, and SH-(CH_2_)_6_-5′-TTT TTT TTT TTT-3′ for probe <II>. Also, in the probe NPs modified with random sequence DNA (PrR): 5′-ATG CTC AAC TCT-3′-(CH_2_)_6_-SH for probe <I>, and SH-(CH_2_)_6_-5′-TCT CAA CTC GTA-3′ for probe <II>, respectively. These DNA sequences were synthesized by Life Technologies. The solution was added to 10 mM phosphate buffer (pH 7.0) containing 0.1 M NaCl (1000 μL); the resulting mixture was maintained at 298 K for 40 h, followed by centrifugation for 25 min at 14,000 rpm at ∼278 K in order to remove any unreacted oligonucleotide. After the removal of the 800 μL supernatant, the AuNPs were washed with 10 mM phosphate buffer solution containing 0.1 M NaCl (1000 μL). After another round of centrifugation under the same conditions, the precipitate was dispersed in 10 mM phosphate buffer containing 0.3 M NaCl (1000 μL). The AuNP modified by the 3′-terminally thiolated DNA was denoted as probe <I>, while the AuNP modified by the 5′-terminally thiolated DNA was denoted as probe <II>. The surface density of the DNA on a probe NP was estimated as 48 pmol/cm^2^. UV-vis spectrometer (V-630BIO, JASCO Corporation) was used for the estimation of the density of DNA on each probe NP and observation of spectral change by the natural hybridization of probe NPs and target DNA.

For the proof-of-principle experiment, a simple thymine base sequence was considered adequate: probe <I> was modified with 5′-TTTTTTTTTTTT-3′-(CH_2_)_6_-SH and probe <II> was modified with SH-(CH_2_)_6_-5′-TTTTTTTTTTTT-3′, respectively (PrT). As target DNA molecules, four types of single-strand 24-base DNAs were used: complementary (AAAA): 5′-AAAAAAAAAAAAAAAAAAAAAAAA-3′; half-mismatched (AATT): 5′-AAAAAAAAAAAATTTTTTTTTTTT-3′, alternate sequence (TATA): 5′-TATATATATATATATATATATATA-3′, and perfectly mismatched (TTTT): 5′-TTTTTTTTTTTTTTTTTTTTTTTT-3′. Furthermore, the random sequence DNA molecules were also used for the comparison experiment with the different complementarity; 5′-ATG CTC AAC TCT-3′-(CH_2_)_6_-SH for probe <I>, SH-(CH_2_)_6_-5′-TCT CAA CTC GTA-3′ for probe <II>, respectively (PrR). As a target DNA, 5′- AGA GTT GAG CAT TAC GAG TTG AGA -3′ was used as the complementary DNA (RAN-C) forming 10 G-C pairs with probe <I> and <II>.

### Numerical Simulations

To evaluate the optical spectra and LIF exerted on the probe NPs, we used our developed “Light-induced Molecular Recognition Metropolis Method (LMRM)” based on the stochastic method under the self-consistently determined optical response field^26^,^27^. The response field **E** and induced polarization **P** were evaluated by solving the relevant Maxwell equations using a discretized integral with spherical cells (DISC)^26^ as follows:


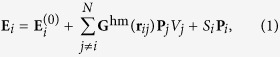



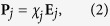


where AuNPs were modeled with spherical cells, *N* is the number of cells (probe NPs), 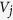 is the volume of each cell, 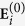 is the electric field component of an incident optical field, **G**^hm^ is the Green’s function in a homogeneous medium, and *χ*_*j*_ is the electric susceptibility. The integral 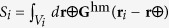 for *i* = *j* as the self-term in equation (1) was calculated. By substituting the obtained **E** and **P** as solutions of the simultaneous equations (1) and (2) into the general expression of LIF^32^.


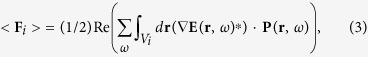


we can evaluate the gradient force, dissipative force, and interparticle LIF. The total light momentum transfer rate is proportional to the extinction. The extinction spectrum of the total system can be calculated by evaluating the sum of the radiation pressure 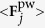 on all of the nanostructures under irradiation of a propagating plane wave as 
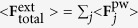
, which is the sum of scattering and absorption forces. Finally, the total scattering spectra of the assembled probe NPs via target DNA can be obtained by evaluating 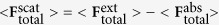, where 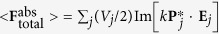 is the absorption component. In addition, Drude electric susceptibility was used to determine the 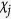 of AuNPs whose parameters were determined from the experimental results and the optical constants published by Johnson and Christy^33^. The focused incident light was assumed to be a Gaussian beam.

In order to determine the final configuration of probe NPs and target DNA (see Supplementary Movies S1 and S2), we used the Monte Carlo simulation assuming self-consistently determined LIF with Equations (1)–(3)^27^,^34^. The variation of optical potential is given as 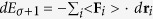 after the random positional changes of probe NPs during the *σ*th and (*σ* + 1)th steps. If 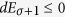 is satisfied, the (*σ* + 1) th state is always adopted since the energy of this state is lower than that of the *σ*th step. On the other hand, if 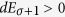 is satisfied, the (*σ* + 1) th state is adopted with the probability of 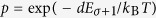 or the configuration does not change since the energy of this state is higher than the *σ*th step. By repeating these procedures, we can find the more stable spatial configuration of assembled AuNPs.

The model for specific binding of probe and target DNA is shown in Fig. S6. Terminally (3′- and 5′)-thiolated DNA molecules on AuNPs are modeled by the semi-spheres to indicate the radius of rotation in a single strand probe DNA, while the target DNA are modeled by the different colored two spheres to express the specific binding with the probe DNA, where red and blue colors show the bindable combination of probe and target DNA. The binding energy of hybridization is given by the interaction energy per base pair comprising the double stranded DNA (*ε*_hbp_) to express the difference of the number of hydrogen bonds between adenine and thymine or between guanine and cytosine. The total interaction energy linearly increases as the number of coupled base pairs gets larger according to the decrease of distance between a target DNA and the surface of a AuNP (*r*). *ε*_hbp_ is assumed to be a few *k*_B_*T* under the coarse grained hybridization model similarly to ref. 35.

## Additional Information

**How to cite this article:** Iida, T. *et al*. Submillimetre Network Formation by Light-induced Hybridization of Zeptomole-level DNA. *Sci. Rep.* **6**, 37768; doi: 10.1038/srep37768 (2016).

**Publisher's note:** Springer Nature remains neutral with regard to jurisdictional claims in published maps and institutional affiliations.

## Supplementary Material

Supplementary Movie S1

Supplementary Movie S2

Supplementary Movie S3

Supplementary Movie S4

Supplementary Movie S5

Supplementary Movie S6

Supplementary Information
