# Supplementary material for: Submillimetre Network Formation by Light-induced Hybridization of Zeptomole-level DNA
Source: Sci Rep. 2016 Dec 5;6:37768. doi: 10.1038/srep37768 (PMC5137144; doi:10.1038/srep37768)
Supplement: Supplementary Information [file srep37768-s7.pdf]

SUPPLEMENTARY INFORMATION

Submillimetre Network Formation by Light-induced Hybridization of Zeptomole-level DNA

Takuya Iida[1,2*,#], Yushi Nishimura[1,2,#], Mamoru Tamura[1,2], Keisuke Nishida[2,3], Syoji Ito[4], and Shiho Tokonami[2,3,*,#]

[1] Department of Physical Science, Graduate School of Science, Osaka Prefecture University, Sakai, Osaka 599-8570, Japan

[2] Nanoscience and Nanotechnology Research Center, Osaka Prefecture University, 1-2, Gakuencho, Nakaku, Sakai, Osaka 599-8570, Japan

[3] Department of Applied Chemistry, Graduate School of Engineering, Osaka Prefecture University, Sakai 599-8570, Japan

[4] Division of Frontier Materials Science, Graduate School of Engineering Science, Osaka University, Toyonaka, Osaka 560-8531, Japan

*Correspondence to: t-iida@p.s.osakafu-u.ac.jp (T.I.); tokonami@chem.osakafu-u.ac.jp (S.T.)

#These authors contributed equally to this work.

Movie Legends:

Please see also the detail in the main text.

Movie S1. Simulation result of dynamics of probe nanoparticles (NPs) and complementary DNA under the self-consistently evaluated light-induced force (LIF) (corresponding to Figure 2b in the main text).

Movie S2. Simulation result of dynamics of probe NPs and mismatched DNA under the self-consistently evaluated LIF (corresponding to Figure 2c in the main text).

Movie S3. Real-time dynamics of probe NPs and complementary DNA (AAAA; 100 pM) under laser irradiation. Recording started at the same time as laser irradiation (corresponding to Figure 3a in the main text and Figure S1a).

Movie S4. Real-time dynamics of probe NPs and half-mismatched DNA (AATT; 100 pM) under laser irradiation. Recording started at the same time as laser irradiation (corresponding to Figure S1b).

Movie S5. Real-time dynamics of probe NPs and alternate-sequence DNA (TATA; 100 pM) under laser irradiation. Recording started at the same time as laser irradiation (corresponding to Figure S1c).

Movie S6. Real-time dynamics of probe NPs and mismatched DNA (TTTT; 100 pM) under laser irradiation. Recording started at the same time as laser irradiation (corresponding to Figure 3a in the main text and Figure S1d).

Supplementary Figures:

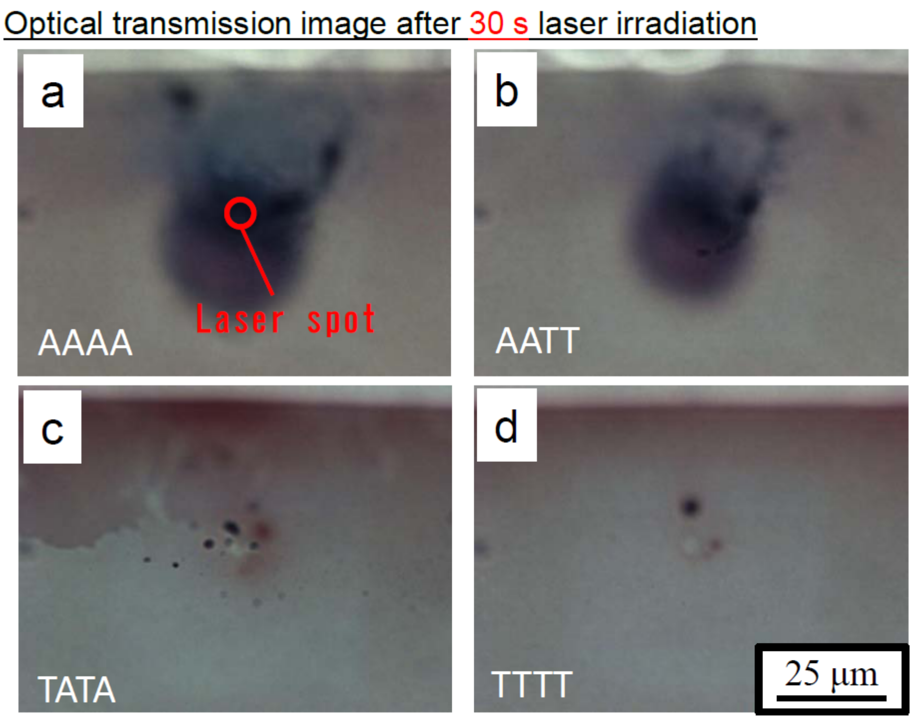

Fig. S1. a – d, Optical transmission images at 30 s after laser irradiation for each sequence of target DNA and T-sequence probe NPs. Laser wavelength is 1064 nm, and input power 0.2 W at the laser source.

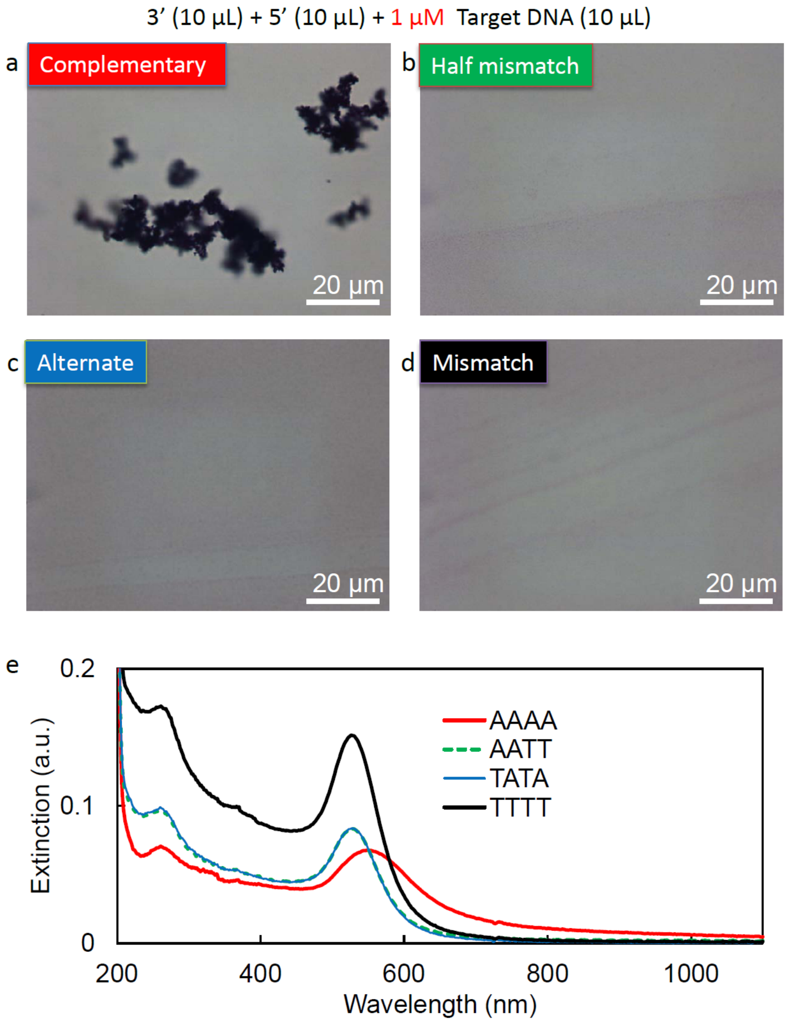

Fig. S2. a-d, Optical transmission images on the glass substrate of probe NPs 3 h after adding target DNA (1 μM). **e,** Extinction spectra of probe NPs in suspension after adding target DNA, observed by UV-vis spectrometry in bulk suspension.

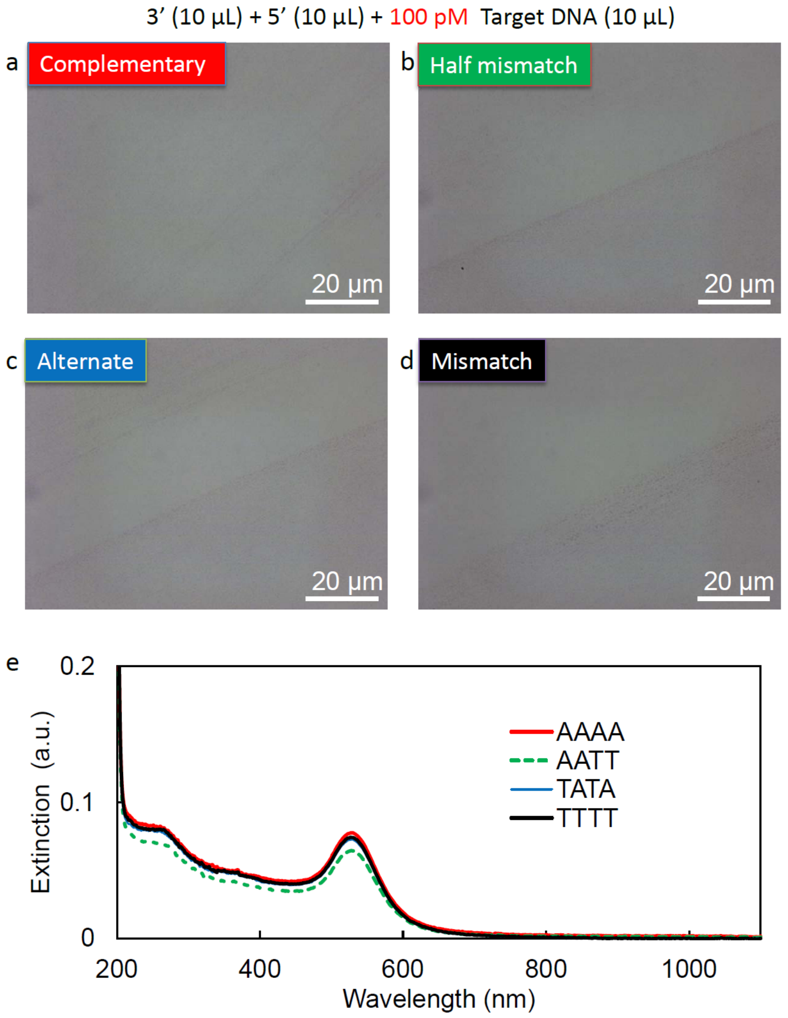

Fig. S3. a-d, Optical transmission images of probe NPs on the glass substrate 3 h after adding target DNA (100 pM). **e,** Extinction spectra of probe NPs in suspension after adding target DNA.

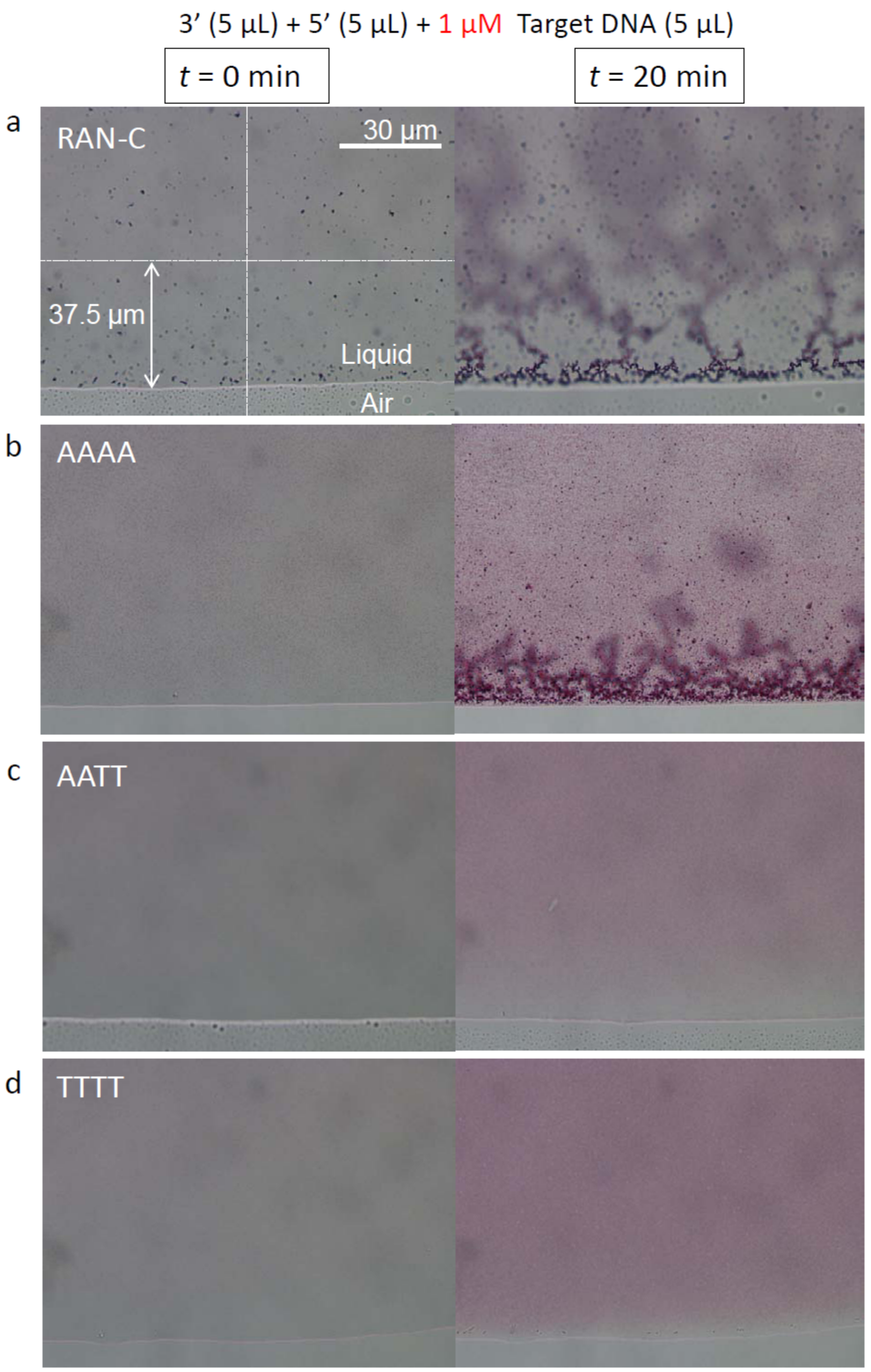

Fig. S4. a-d, Optical transmission images of dispersed liquid droplets of probe NPs near the air-liquid interface on the glass substrate 20 minutes after adding different target DNA (1 μM).

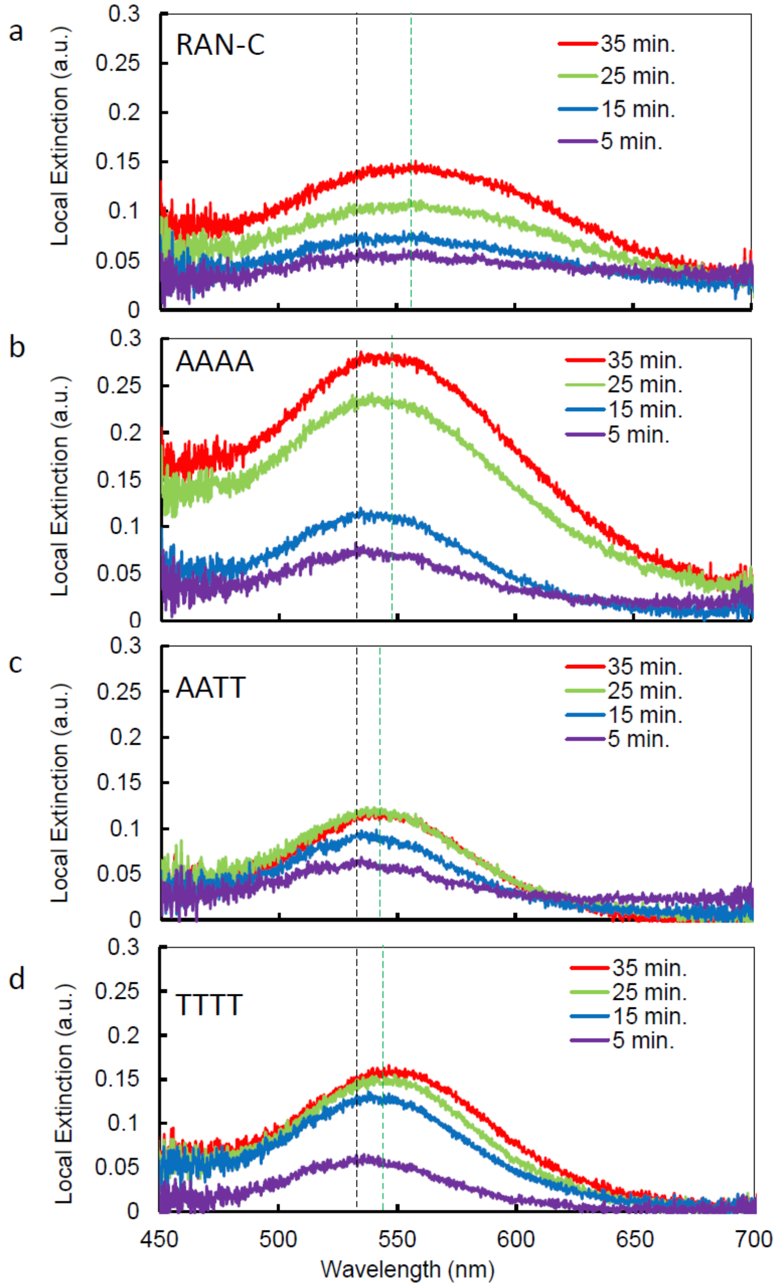

Fig. S5. a-d, Local extinction spectra of dispersed liquid droplets of probe NPs near the air-liquid interface after adding different target DNA (1 μM), which corresponds to Fig. S4.

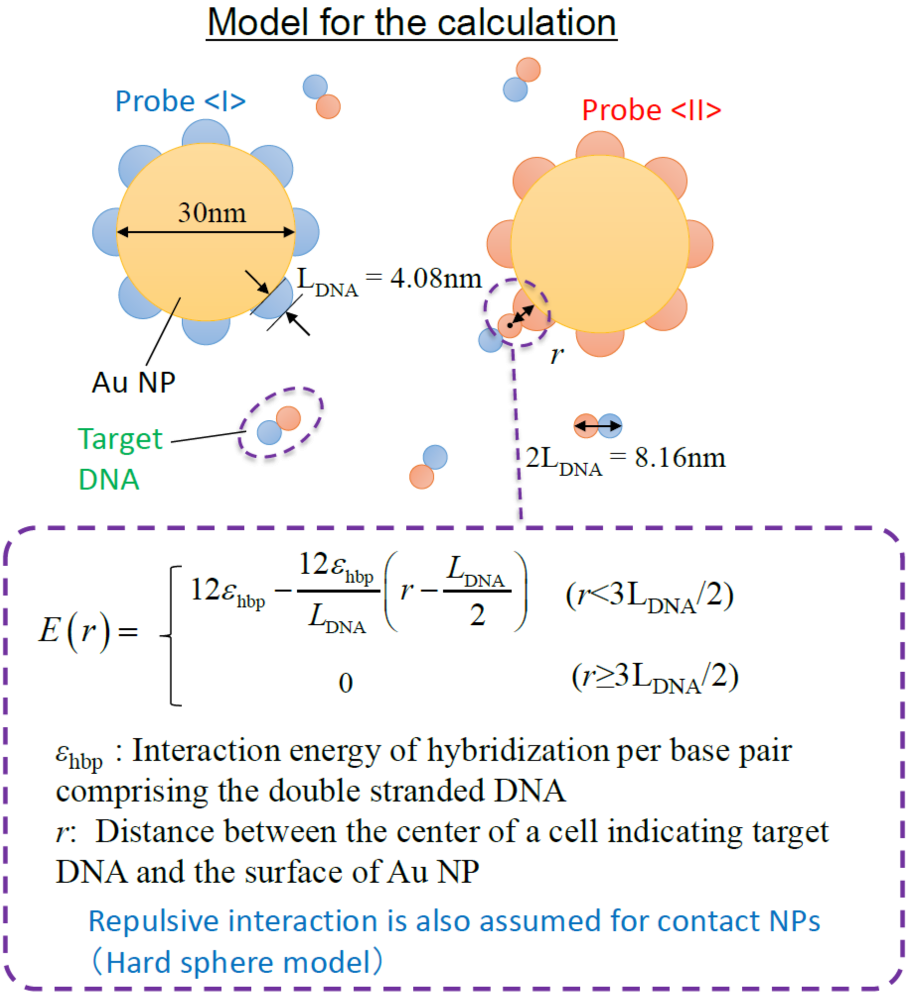

Fig. S6. Model for the calculation of optically accelerated hybridization of DNA on AuNPs.

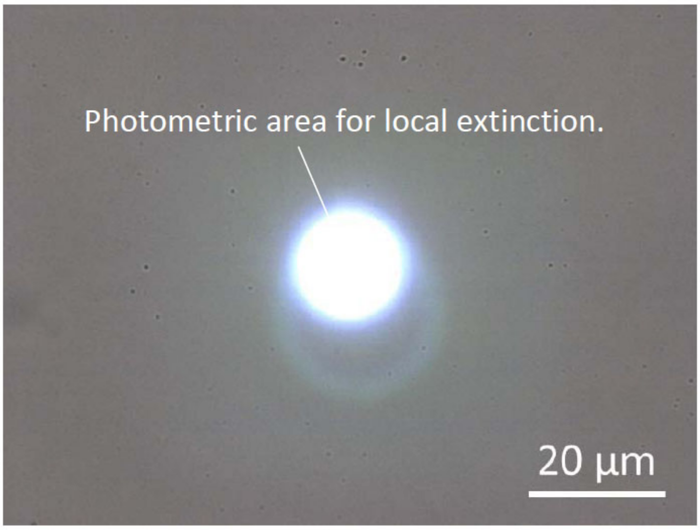

Fig. S7. Photometric area for the observation of local extinction spectra.
